# Downregulation of Lgals3 Alleviates Inflammatory Response and Apoptosis in a Mouse Model of Cerebral Ischemia/Reperfusion Injury

**DOI:** 10.1002/brb3.71700

**Published:** 2026-08-20

**Authors:** Zhaodan Gan, Haifang Lai, Yun Yang, Jun Lu, Wen Zhang, Jianqiu Xiao, Guangxu Xu

**Affiliations:** ^1^ Department of Rehabilitation Medicine The First Affiliated Hospital of Nanjing Medical University Nanjing China; ^2^ Department of Rehabilitation Medicine Ruijin Hospital Shanghai Jiao Tong University School of Medicine Shanghai China; ^3^ School of Basic Medicine Nanjing Medical University Nanjing China

**Keywords:** cerebral IR injury, neuroinflammation, neuronal apoptosis, TLR4/MyD88/NF‐κB pathway

## Abstract

**Purpose:**

The present work investigated the expression and function of Lgals3 in cerebral ischemia/reperfusion (cerebral IR) injury.

**Methods:**

Differentially expressed genes in cerebral IR were analyzed by the Gene Expression Omnibus (GEO) microarray. A middle cerebral artery occlusion (MCAO) mouse model was constructed. Adeno‐associated virus serotype 9 vector carrying Lgals3 shRNA was administered to mice via tail vein injection. Four weeks after virus administration, mice were subjected to MCAO modeling. Neurological function was reflected by evaluating the neurological deficit score. To assess infarct size and neuronal apoptosis, triphenyl tetrazolium chloride (TTC) staining and TUNEL assay were performed. The concentrations of IL‐6, TNF‐α, IL‐4, and IL‐1β in brain tissues were measured using enzyme‐linked immunosorbent assay (ELISA). Co‐immunoprecipitation (Co‐IP) was performed to examine the interaction between Lgals3 and TLR4. In addition, real‐time quantitative reverse transcription‐polymerase chain reaction (qRT‐PCR) and Western blotting were employed to analyze gene and protein expression levels.

**Results:**

GEO bioinformatics analysis revealed that Lgals3 expression was significantly upregulated in cerebral IR. MCAO mouse model was successfully established, and Lgals3 was markedly upregulated in brain tissues of MCAO mice. Lgals3 knockdown reduced infarct volume, improved neurological deficit scores, and inhibited neuronal apoptosis, accompanied by decreased levels of pro‐inflammatory cytokines (IL‐6, TNF‐α, and IL‐1β) and increased levels of anti‐inflammatory cytokine IL‐4 in brain tissues of MCAO mice. Lgals3 silencing inhibited the activation of the TLR4/MyD88/NF‐κB pathway, as evidenced by downregulated expression of TLR4, MyD88, p‐p65, and p65. Co‐IP experiment confirmed a direct interaction between Lgals3 and TLR4.

**Conclusion:**

Lgals3 knockdown alleviates neuroinflammation and neuronal apoptosis in brain tissues of mice with cerebral IR injury. The present findings indicate that Lgals3 participates in the progression of cerebral IR injury, and may serve as a potential intervention target for ameliorating cerebral IR damage.

## Graphical abstract

Mechanistic study of Lgals3 gene exacerbating cerebral ischemia‐reperfusion injury via the TLR4/MyD88/NF‐κB pathway.

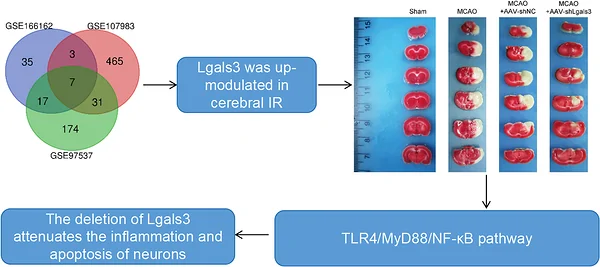

## 1 Introduction

Ischemic stroke is caused by the occlusion of cerebral arteries, accounting for approximately 80% of all stroke cases (Y. Wang et al. 2025; Iadecola and Anrather 2011). Rapid restoration of cerebral perfusion is currently the first‐line therapeutic strategy for ischemic stroke, represented by intravenous thrombolysis (Bangad et al. 2023). However, this process can lead to further injury to the brain tissue (known as cerebral IR injury), resulting in additional neurological dysfunction and grim negative prognostic impact on patients (Bindal et al. 2024; H. Wang et al. 2023). It has been revealed that severe cerebral IR injury can induce the dysfunction of multiple organs, permanent disability, and even death (Liang et al. 2021; Chamorro et al. 2012). Hence, finding effective regimens for the treatment of cerebral IR injury is extremely essential to improve the prognosis of ischemic stroke.

Lgals3 (also known as galectin‐3 [Gal3]) is one of the key members of the galectin family, which has been discovered to play potential functions in multiple diseases, such as rheumatoid arthritis, necrotizing enterocolitis, and several common cancers (Wu et al. 2024; Z. Ye et al. 2025; D. Cheng, Lian et al. 2024; Lu and Weiser‐Evans 2022). Further, Lgals3 has been suggested to be linked to the myocardial IR injury, as its down‐modulation alleviated the myocardial IR injury of mouse (Rong et al. 2020). Mo et al. (2019) performed RNA sequencing analysis and discovered the indispensable role of Lgals3 in IR‐trigger myocardial injury; they demonstrated the upregulated Lgals3 in myocardial IR injury; intriguingly, the inhibition of Lgals3 reduced the infarct area, relieved the myocardial tissue damage and cardiomyocyte apoptosis in myocardial IR injury animal model. Moreover, the knockdown of Lgals3 has been revealed to ameliorate the inflammatory response in the myocardial IR injury cell model (M. Zhang et al. 2020). Recently, evidence has emerged that Lgals3 is associated with the regulation of the cerebral IR injury; the blockage of Lgals3 exerted the neuroprotective effect after ischemic stroke, possibly by suppressing the activation of inflammatory responses in microglia (Cui et al. 2022). However, relevant evidence regarding the role of Lgals3 in cerebral IR injury remains limited.

In our preliminary study, we found that upregulated Lgals3 in mice after cerebral IR, based on the data analysis retrieved from the Gene Expression Omnibus (GEO) database. Thereby, this study established a cerebral IR injury mouse model and an Lgals3‐knockdown cerebral IR injury mouse model, to explore the specific role of Lgals3 in the progression of cerebral IR injury.

## 2 Materials and Methods

### 2.1 Microarray‐Based Gene Expression Analysis Based on the GEO Database

The cerebral IR‐associated microarray data were downloaded from the GEO (https://www.ncbi.nlm.nih.gov/geo) database, including GSE97537 (12 Sprague‐Dawley rats, with 7 subjected to MCAO and 5 undergoing sham operation, and comparing mRNA expression in the brain between these two groups.) GSE107983 (12‐ to 14‐week‐old male wild‐type or toll‐like receptor 4 (TLR4)−/− mice subjected to transient middle cerebral artery occlusion [MCAO] followed by 72 h of tMCAO/R or sham surgery under isoflurane anesthesia, with RNA extracted from ex vivo sorted microglia of the ipsilateral cortex and profiled via microarray) and GSE166162 (young and aged rats subjected to MCAO or sham operation, where differences in mRNA expression between MCAO‐treated young and aged rats were analyzed by subtracting baseline differences observed in sham‐operated counterparts, isolating genes specifically dysregulated by ischemic injury).

We confirmed that all differential expression analyses were adjusted for multiple comparisons using the Benjamini–Hochberg FDR procedure (FDR < 0.05). Volcano plot analysis and the limma package in R were used to identify differentially expressed genes. Genes with *p* < 0.05 and |log fold change| > 2 were selected as statistically significant. De‐batch correction and PCA analysis were implemented for the preprocessing of data.

### 2.2 Construction of Middle Cerebral Artery Occlusion Mouse Model

Twenty‐four male C57BL/6J mice aged 6–8 weeks (body weight 20–25 g) were purchased from the Shanghai Experimental Animal Center, Chinese Academy of Sciences. The animals were maintained in a specific pathogen‐free (SPF) facility under controlled conditions (22°C, 12‐h light/dark cycle) with ad libitum access to food and water. All experimental procedures were approved by the Animal Ethics Committee (CMEJK023).

The 24 mice were divided into four groups: Sham group, MCAO group, MCAO + AAV‐shNC group, and MCAO + AAV‐shLgals3 group, with six mice per group. The construction of MCAO model was carried out on mice of the MCAO group, the MCAO + AAV‐shNC group, and the MCAO + AAV‐shLgals3 group. The surgical procedure was as follows (G.‐Y. Wang, Wang, et al. 2020): mice were anesthetized with 2% isoflurane, and then underwent the induction of cerebral ischemia by ligating the right middle cerebral artery with 6–0 nylon monofilament; the suture was withdrawn after 1 h of occlusion, and mice underwent reperfusion for 24 h. The same surgical procedure was implemented on mice of the Sham group, but without the ligation. After cerebral IR, mice were subjected to the examination of neurological deficits, deeply anaesthetized with 5% isoflurane, and then euthanized to collect the whole brain tissues.

### 2.3 Adeno‐Associated Virus Serotype Vectors Infection of Mice

Adeno‐associated virus serotype (AAV) vectors carrying either Lgals3 shRNA or shRNA negative control (NC) were provided by GeneChem (Shanghai, China). Before use, AAV vectors were prepared into 3 × 10^11^ genome copies per 100 µL phosphate‐buffered saline. Mice of the MCAO + AAV‐shNC group and the MCAO + AAV‐shLgals3 group were injected intravenously with AAV vectors carrying either shNC or Lgals3 shRNA through tail vein at a dose of 1.5 × 10^11^ genome copies per mouse (G.‐Y. Wang, Wang, et al. 2020). Four weeks after infection, mice of the MCAO + AAV‐shNC group and the MCAO + AAV‐shLgals3 group underwent the cerebral IR surgery as described above.

### 2.4 Neurological Deficit Score

The evaluation of mice's neurological deficits was performed by Longa's method as previously described (G.‐Y. Wang, Wang, et al. 2020). The neurological deficit score consists of 0, 1, 2, 3, and 4 points. Mice without neurological deficits were evaluated with a score of 0, and a score of 4 meant the most severe neurological deficit. A higher neurological deficit score indicated more severe neurological damage.

### 2.5 Triphenyl Tetrazolium Chloride Staining

Brain tissues (ischemic penumbra) of mice were prepared into coronal sections (2 mm) for incubation with 2% triphenyl tetrazolium chloride (TTC) solution in darkness (for 20 min at 37°C). The residual TTC solution (Solarbio, Beijing, China) was rinsed off with phosphate‐buffered saline. After being immobilized with 4% paraformaldehyde for 2 h, the sections were photographed to measure the infarct volume by (total infarct volume/total brain volume) × 100% (Y. Zhang et al. 2019). The ischemic region appeared white upon staining, and its quantification was conducted using Image‐Pro Plus 6.0 (Media Cybernetics, Bethesda, MD, USA).

### 2.6 TUNEL Assay

The apoptotic neurons in mouse brain tissues (ischemic penumbra and the infarct core area) were detected by using the TUNEL assay kit (Roche, Shanghai, China) according to the instructions. The brain tissues of mice were paraffin‐embedded and prepared into sections (4 µm). After being dewaxed and hydrated in xylene and graded alcohol, the sections were treated with TUNEL reaction buffer for 1 h at 37°C. The residual buffer was removed with phosphate‐buffered saline. The TUNEL‐positive cells (apoptotic neurons) were photographed and counted under a light microscope (Olympus, Tokyo, Japan).

### 2.7 Enzyme‐Linked Immunosorbent Assay

The brain tissues (peri‐infarct region of the ischemic hemisphere) of mouse were homogenized on ice with lysis buffer. Then the mixture was centrifuged (for 10 min at 3000 × *g* and 4°C) to harvest the supernatant. The levels of pro‐inflammatory factors (IL‐6, TNF‐α, and IL‐1β) and anti‐inflammatory factor (IL‐4) in the supernatant were detected by using the relevant commercial enzyme‐linked immunosorbent assay (ELISA) kits. The examination process was carried out followed by the instuctions of kits. The mouse IL‐6 assay kit (K4144‐100), TNF‐α assay kit (K1051‐100), IL‐4 assay kit (KA3063), and IL‐1β assay kit (K4795‐100) were all purchased from AmyJet Scientific (Wuhan, China).

### 2.8 Co‐Immunoprecipitation (Co‐IP)

Lysates were prepared in ice‐cold lysis buffer, centrifuged, and pre‐cleared. Anti‐Lgals3 antibody was added to lysates for overnight rotation at 4°C (IgG control included). Pre‐equilibrated Protein A/G beads captured complexes during 2–4 h incubation, followed by 4–5 wash cycles. Boiled elution in SDS buffer enabled sodium dodecyl sulfate‐polyacrylamide gel electrophoresis (SDS‐PAGE)/Western blotting with anti‐TLR4 antibody, comparing Input, IP, and IgG groups.

### 2.9 Real‐Time Quantitative Reverse Transcription‐Polymerase Chain Reaction (qRT‐PCR)

Total RNA was extracted from the peri‐infarct region of the ischemic hemisphere in mouse brain tissue using TRIzol reagent (Solarbio, China) according to the manufacturer's instructions. Reverse transcription reaction was implemented to synthesize cDNA templates via using the PrimeScript RT Reagent Kit (Takara, Shiga, Japan) according to the directions. qPCR was executed with SYBR Green dye (Takara, Shiga, Japan) on the ABI7500 Real‐Time PCR System (Applied Biosystems, Foster City, CA, USA). The reaction parameters of qPCR were 95°C for 30 s, 60°C for 34 s, and 72°C for 30 s, with 40 cycles. The primers were: Lgals3, sense: 5'‐GGCCACTGATTGTGCCTTAT‐3'; antisense: 5'‐GAAGCGTGGGTTAAAGTGGA‐3'. Glyceraldehyde‐3‐phosphate dehydrogenase (GAPDH), sense: 5'‐GCGAGATCCCTCCAAAATCAA‐3'; antisense: 5'‐GTTCACACCCATGACGAACAT‐3'. The relative Lgals3 mRNA expression was calculated by the 2^−ΔΔCt^ method (H. Wang, Zheng, et al. 2020) and normalized to GAPDH.

### 2.10 Western Blot

RIPA lysis buffer (Solarbio, Beijing, China) was added into the peri‐infarct area of the ischemic hemisphere in mouse brain tissue to extract the total proteins following the manufacturer's instructions. After the determination of total protein concentration, 10% SDS‐PAGE was executed for the separation of total proteins. Subsequently, the proteins were transferred to the polyvinylidene fluoride (PVDF) membranes to experience the blockage by 5% skimmed milk (for 1 h at room temperature). The proteins were sequentially subjected to primary antibody incubation (a dilution of 1:1000; for 12 h at 4°C) and secondary antibody reaction (a dilution of 1:2000; for 2 h at room temperature). The primary antibodies were: rabbit anti‐Lgals3 (PAB14868, AmyJet Scientific, Wuhan, China), rabbit anti‐Bax (ab262929, Abcam, Cambridge, UK), rabbit anti‐Cleaved caspase‐3 (ab2302, Abcam, Cambridge, UK), rabbit anti‐Bcl‐2 (A‐AP52218, AmyJet Scientific, Wuhan, China), mouse anti‐Cleaved poly‐ADP‐ribose polymerases (PARP) (ABM40132, AmyJet Scientific, Wuhan, China), rabbit anti‐TLR4 (ab150583, Abcam, Cambridge, UK), rabbit anti‐MyD88 (ab2064, Abcam, Cambridge, UK), rabbit anti‐p‐p65 (A‐AP50078, AmyJet Scientific, Wuhan, China), rabbit anti‐p65 (ab16502, Abcam, Cambridge, UK) and rabbit anti‐GAPDH (ABP50152, AmyJet Scientific, Wuhan, China). The secondary antibodies were horseradish peroxidase‐labeled goat anti‐rabbit (ab6721, Abcam, Cambridge, UK) or goat anti‐mouse (ab6808, Abcam, Cambridge, UK) secondary antibodies. Enhanced chemiluminescence (ECL) kit (Yeasen Biotechnology, Shanghai, China) was employed to develop the protein bolts. The proteins were quantified by detecting the gray values of protein bands via employing the Image J software (version 1.46r, ImageJ, NIH, Bethesda, MD, USA). The relative expression of proteins was normalized against GAPDH.

### 2.11 Statistical Analysis

Experiments were performed with *N* = 3 independent biological replicates. All data are presented as mean ± standard deviation. GraphPad Prism 9.0 software (GraphPad Software, San Diego, CA, USA) was used for statistical analysis. Before parametric statistical testing, the normality of data distribution and homogeneity of variance were routinely evaluated. Two‐tailed unpaired Student's *t*‐test was applied for pairwise comparisons, and one‐way ANOVA followed by Tukey's post hoc test was used for multiple group comparisons. A *p*‐value less than 0.05 was considered statistically significant.

## 3 Results

### 3.1 Lgals3 Was Upregulated in Cerebral IR Based on GEO Analysis

**FIGURE 1:**
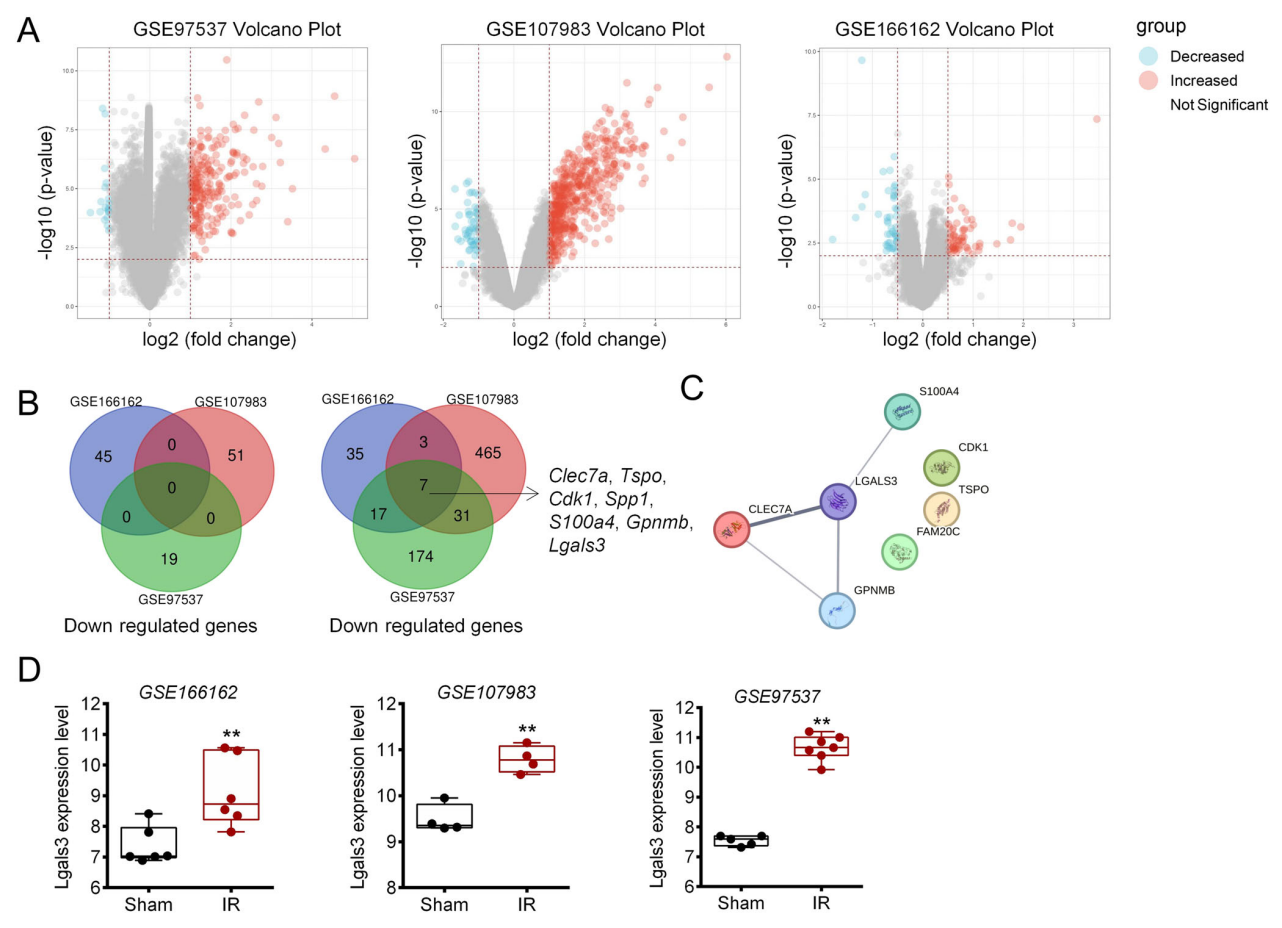
Lgals3 was upregulated in cerebral IR based on GEO analysis. (A) The differentially expressed genes were analyzed by volcano plot based on data retrieved from the GEO database, including the GSE97537, GSE107983, and GSE166162 datasets. (B) Venn diagram was plotted to screen for overlapping differentially expressed genes in cerebral IR, and seven upregulated genes were revealed to overlap, including Clec7a, Tspo, Cdk1, Spp1, S100a4, Gpnmb, and Lgals3. (C) Enrichment analysis was performed on the seven overlapping genes by using the STRING platform. Lgals3 was proven to be the center of enrichment among the seven overlapping genes. (D) Lgals3 was revealed to be upregulated in cerebral IR model based on the GSE97537, GSE107983, and GSE166162 datasets. **p < 0.01 vs. Sham group. IR, ischemia‐reperfusion; GEO, Gene Expression Omnibus.

Cerebral IR‐related microarray data were retrieved from the GEO database, including datasets GSE97537, GSE107983, and GSE166162. DEGs were screened by volcano plot. As a result, 230 upregulated genes and 45 downregulated genes were found in the GSE97537 dataset; 506 upregulated genes and 19 downregulated genes were discovered in the GSE107983 dataset; 63 upregulated genes and 45 downregulated genes were discovered in the GSE166162 dataset (Figure 1A). Batch effect correction and PCA analysis were carried out to preprocess the data, which is shown in Figure S1A,B. We plotted Venn diagram for the differentially expressed genes in cerebral IR. Seven upregulated genes overlapped, including Clec7a, Tspo, Cdk1, Spp1, S100a4, Gpnmb, and Lgals3, while no overlapping downregulated genes were detected (Figure 1B). Subsequently, functional enrichment analysis of these seven overlapping genes was conducted using the STRING database. Network analysis revealed that Lgals3 served as the core hub gene among the seven candidates (Figure 1C). We further analyzed the expression of Lgals3 in cerebral IR model based on GSE97537, GSE107983, and GSE166162 datasets. The results demonstrated that Lgals3 expression was significantly elevated in cerebral IR model (*p* < 0.01) (Figure 1D). Collectively, these findings confirmed that Lgals3 is markedly upregulated following cerebral IR. We next investigated its biological roles in cerebral IR injury in subsequent experiments.

### 3.2 Loss of Lgals3 Reduced Infarct Area and Improved Neurological Function in Mice After Cerebral IR

**FIGURE 2:**
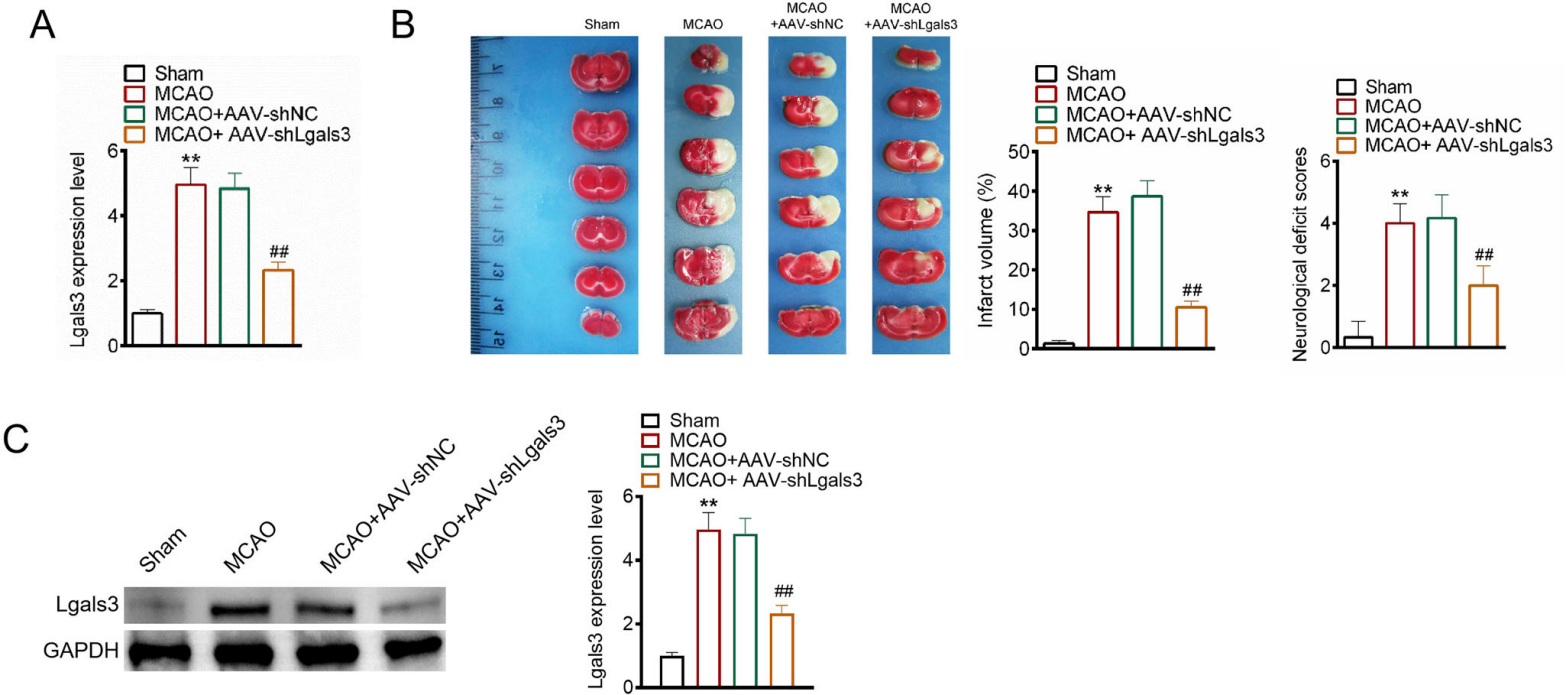
Loss of Lgals3 reduced infarct area and improved neurological function in cerebral IR mice. (A) qRT‐PCR of brain tissues (peri‐infarct region of the ischemic hemisphere) indicated increased Lgals3 mRNA in MCAO mice; AAV9‐Lgals3 shRNA virus delivery reduced Lgals3 mRNA in brain tissues of MCAO mice. (B) TTC staining suggested a reduction in infarct volume in MCAO mice by AAV9‐Lgals3 shRNA virus delivery; neurological function evaluation revealed a decline in neurological deficit score in MCAO mice by AAV9‐Lgals3 shRNA virus delivery. Section angles were optimized for maximal infarction area visualization without affecting volumetric quantification. (C) Western blot of brain tissues (peri‐infarct region of the ischemic hemisphere) illustrated elevated Lgals3 protein in MCAO mice; AAV9‐Lgals3 shRNA virus delivery led to a reduction of Lgals3 protein in the brain tissues of MCAO mice. Data were shown as mean ± SD, *n* = 6. **p < 0.01 vs. Sham group. ##*p* < 0.01 vs. MCAO + AAV‐shNC group. AAV, adeno‐associated virus; IR, ischemia‐reperfusion; MCAO, middle cerebral artery occlusion; qRT‐PCR, quantitative real‐time polymerase chain reaction; SD, standard deviation; shRNA, short hairpin RNA; TTC, 2;3;5‐triphenyltetrazolium chloride.

We established a cerebral IR mouse model by MCAO. Mice were injected with either AAV9 carrying Lgals3 shRNA or AAV9 carrying NC shRNA. qRT‐PCR of brain tissues showed the elevated expression of Lgals3 mRNA in mice of the MCAO group, in contrast to the Sham group (*p* < 0.01); however, a markedly diminished expression of Lgals3 mRNA occurred in mice of the MCAO + AAV‐shLgals3 group, when compared to the MCAO + AAV‐shNC group (*p* < 0.01) (Figure 2A). Mice of the MCAO group exhibited larger cerebral infarct volumes and higher neurological deficit scores than the Sham group (*p* < 0.01). In contrast, smaller infarct volumes and lower neurological deficit scores were observed in mice of the MCAO + AAV‐shLgals3 group, in comparison to the MCAO + AAV‐shNC group (*p* < 0.01) (Figure 2B). Western blot of mouse brain tissues showed a higher expression of Lgals3 protein in the MCAO group than in the Sham group (*p* < 0.01); conversely, a reduction in the expression of Lgals3 protein was detected in brain tissues of mice in the MCAO + AAV‐shLgals3 group, relative to the MCAO + AAV‐shNC group (*p* < 0.01) (Figure 2C). All of these data suggested that upregulated Lgals3 in mice with cerebral IR, and the loss of Lgals3 reduced infarct area and improved neurological function in mice with cerebral IR.

### 3.3 Deletion of Lgals3 Mitigated Neuronal Apoptosis in Mice After Cerebral IR

**FIGURE 3:**
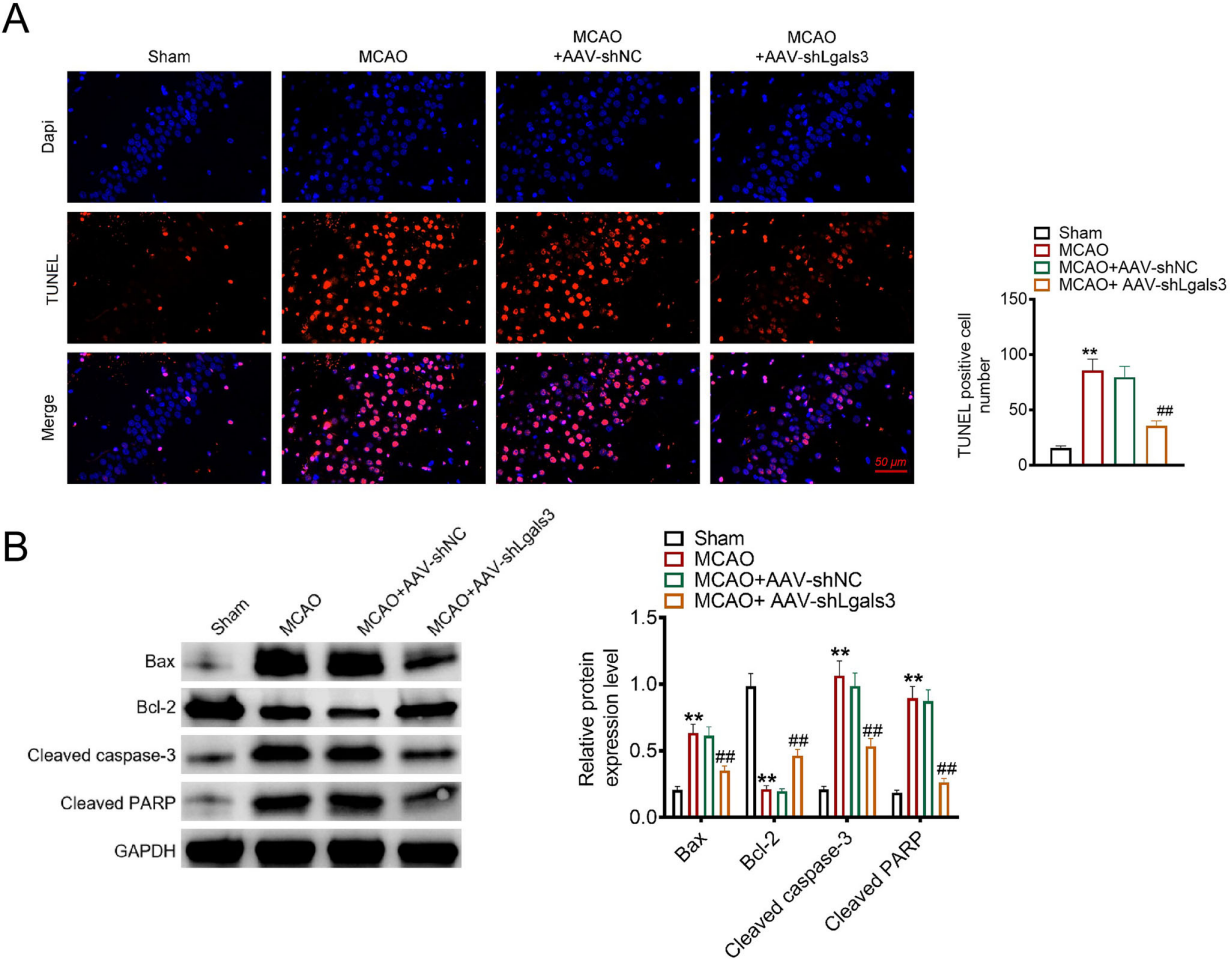
Deletion of Lgals3 mitigated neuronal apoptosis in mice after cerebral IR. (A) TUNEL assay implied that the deletion of Lgals3 suppressed cell apoptosis in brain tissues (ischemic penumbra and the infarct core area) of MCAO mice. (B) Western blot of brain tissues (peri‐infarct region of the ischemic hemisphere) demonstrated that Lgals3 deletion repressed the expression of Bax, Cleaved caspase‐3, and Cleaved PARP proteins, but enhanced the expression of Bcl‐2 protein in MCAO mice. Data were shown as mean ± SD, *n* = 6. ** *p*< 0.01 vs. Sham group. ##*p* < 0.01 vs. MCAO + AAV‐shNC group. IR, ischemia‐reperfusion; MCAO, middle cerebral artery occlusion; PARP, poly(ADP‐ribose) polymerase; SD, standard deviation; TUNEL, terminal deoxynucleotidyl transferase dUTP nick end labeling.

Neuronal apoptosis in mouse brain tissues was assessed via TUNEL staining. The intensified neuronal apoptosis was found in mouse brain tissues of the MCAO group, in comparison to the Sham group (*p* < 0.01); matched to the MCAO + AAV‐shNC group, mitigated cell apoptosis occurred in mouse brain tissues of the MCAO + AAV‐shLgals3 group (*p* < 0.01) (Figure 3A). We further detected the expression of apoptosis‐related proteins in mouse brain tissues by Western blot. The upregulated Bax, down‐modulated Bcl‐2, upregulated Cleaved caspase‐3, and upregulated Cleaved PARP proteins, was observed in brain tissues of mice in the MCAO group, when compared to the Sham group (*p* < 0.01); conversely, in contrast to mice of the MCAO + AAV‐shNC group, decreased Bax, as well as elevated Bcl‐2 protein, decreased Cleaved caspase‐3, and decreased Cleaved PARP proteins, was discovered in mouse brain tissues of the MCAO + AAV‐shLgals3 group (*p* < 0.01) (Figure 3B). Therefore, deletion of Lgals3 could mitigate neuronal apoptosis in brain tissues of mice after cerebral IR.

### 3.4 Silencing of Lgals3 Alleviates Neuroinflammation in Mice After Cerebral IR

**FIGURE 4:**
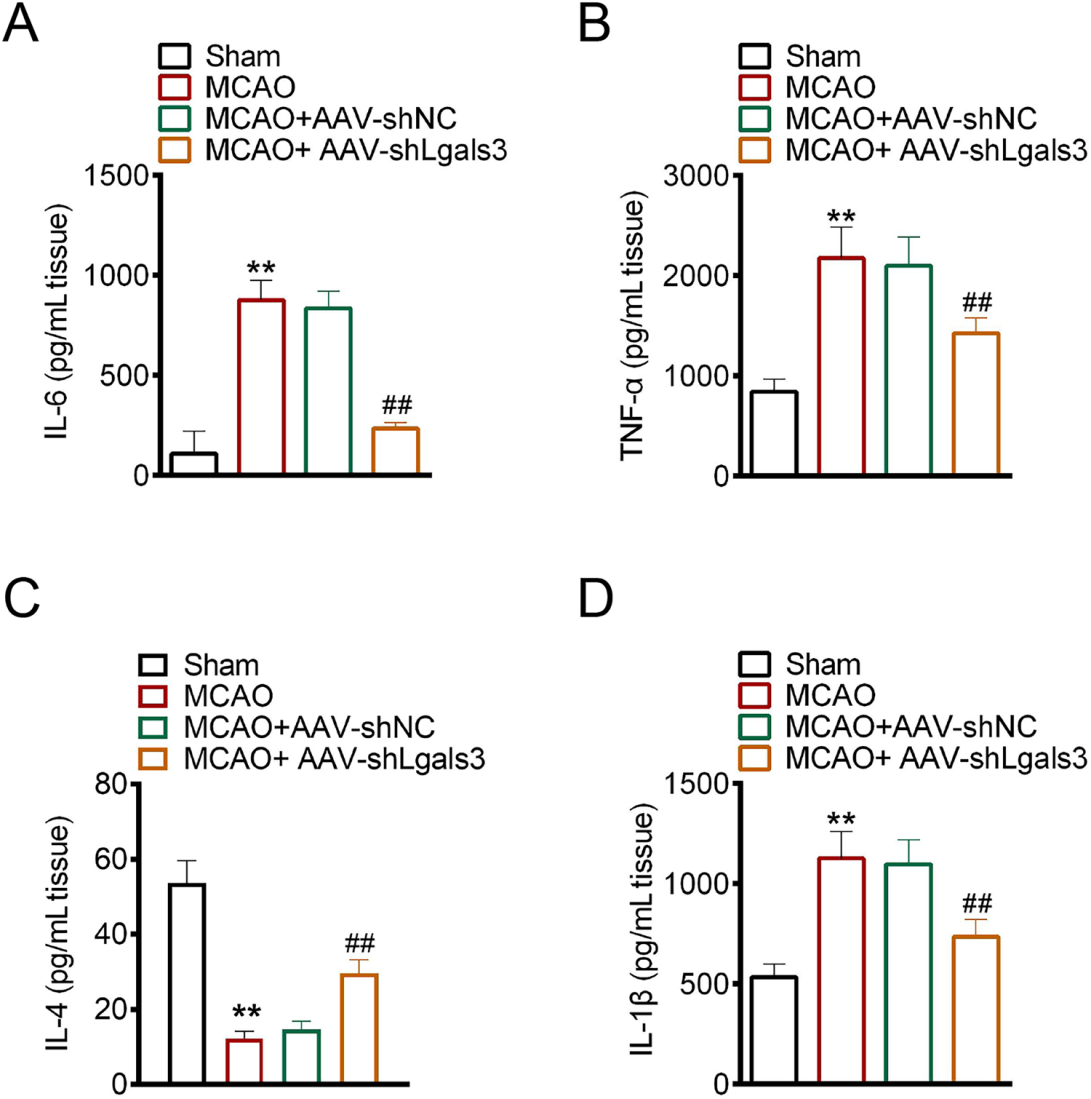
Silencing of Lgals3 alleviates neuroinflammation in mice after cerebral IR. (A–D) ELISA showed enhanced secretion of pro‐inflammatory factors (including IL‐6, TNF‐α, IL‐4, and IL‐1β) in brain tissues (peri‐infarct region of the ischemic hemisphere) of MCAO mice; Lgals3 silencing exerted a suppressive function on the secretion of these pro‐inflammatory factors in brain tissues of MCAO mice. Data were shown as mean ± SD, *n* = 6. **p < 0.01 vs. Sham group. ##*p* < 0.01 vs. MCAO + AAV‐shNC group. ELISA, enzyme‐linked immunosorbent assay; IL, interleukin; IR, ischemia‐reperfusion; MCAO, middle cerebral artery occlusion; SD, standard deviation; TNF‐α, tumor necrosis factor‐alpha.

To explore the role of Lgals3 in the neuroinflammation induced by cerebral IR, we measured the levels of pro‐inflammatory factors (IL‐6, TNF‐α, and IL‐1β) and anti‐inflammatory factor (IL‐4) in mouse brain tissues. As presented in Figure 4A–D, the higher levels of IL‐6, TNF‐α, and IL‐1β and the lower IL‐4 level were found in mouse brain tissues of the MCAO group than in the Sham group (*p* < 0.01); intriguingly, in contrast to the MCAO + AAV‐shNC group, mice of the MCAO + AAV‐shLgals3 group showed lower levels of IL‐6, TNF‐α, and IL‐1β and the higher IL‐4 level in brain tissues (*p* < 0.01). These results implied that Lgals3 knockdown alleviates neuroinflammation in mice with cerebral IR injury.

### 3.5 Lgals3 Silencing Inactivated the TLR4/MyD88/NF‐κB Pathway in Mice After Cerebral IR

**FIGURE 5:**
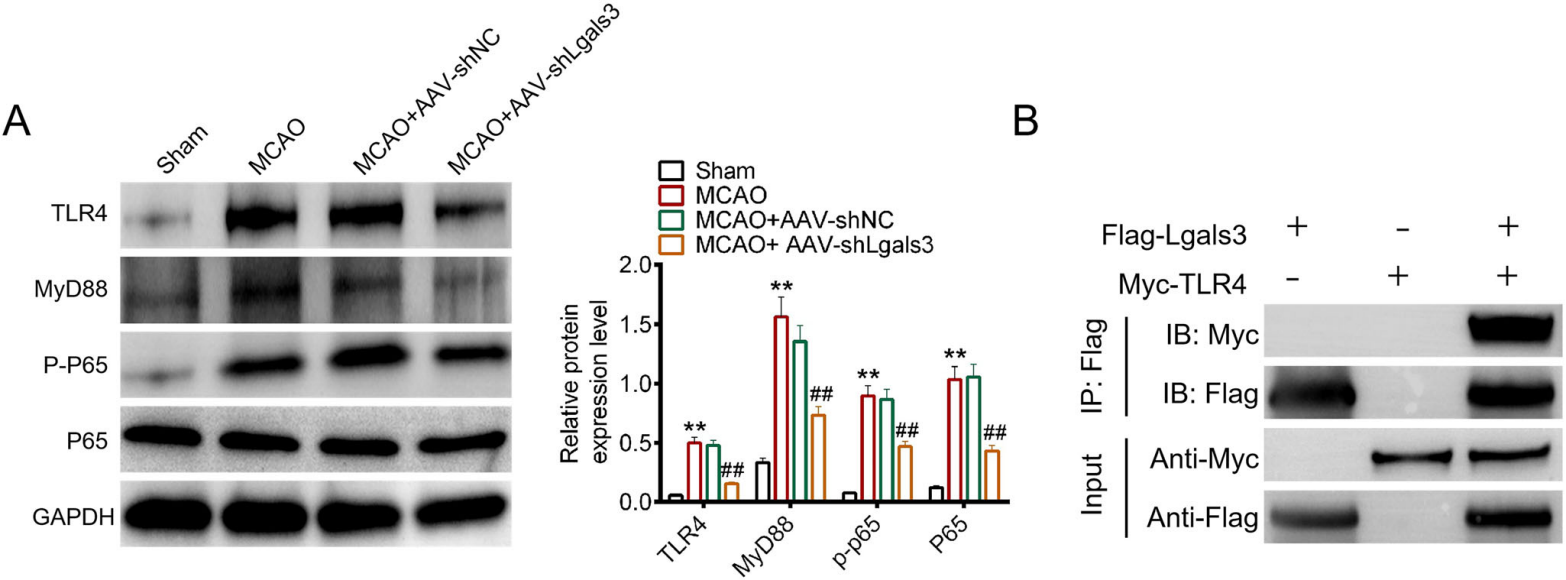
Lgals3 silencing inactivated the TLR4/MyD88/NF‐κB pathway in mice after cerebral IR. (A) Western blot was performed on the brain tissues (peri‐infarct region of the ischemic hemisphere) of mice, and Lgals3 silencing was revealed to inactivate the TLR4/MyD88/NF‐κB pathway in brain tissues of MCAO mice. (B) Co‐IP experiment confirmed a direct interaction. Data were shown as mean ± SD, *n* = 6. **p < 0.01 vs. Sham group. ^##^ *p* < 0.01 vs. MCAO + AAV‐shNC group. IR, ischemia‐reperfusion; TLR4, toll‐like receptor 4; MyD88, myeloid differentiation primary response gene 88; NF‐κB, nuclear factor kappa B; MCAO, middle cerebral artery occlusion; Co‐IP, co‐immunoprecipitation; SD, standard deviation.

We evaluated the activity of the TLR4/MyD88/NF‐κB pathway in mouse brain tissues via Western blot, and the results are presented in Figure 5A. An activated TLR4/MyD88/NF‐κB pathway was found in the brain tissues of mice after cerebral IR, as suggested by the higher expression of TLR4, MyD88, p‐p65, and p65 proteins in the MCAO group than in the Sham group (*p* < 0.01). In addition, mice of the MCAO + AAV‐shLgals3 group expressed lower TLR4, MyD88, p‐p65, and p65 proteins in brain tissues, compared to the MCAO + AAV‐shNC group (*p* < 0.01). Thus, Lgals3 silencing inactivated the TLR4/MyD88/NF‐κB pathway in brain tissues of mice after cerebral IR. Moreover, Co‐IP experiment confirmed a direct interaction between Lgals3 and TLR4 (Figure 5B).

## 4 Discussion

The Lgals3 gene encodes Gal3, an important glycan‐binding protein that functions as a danger signal and inflammatory mediator. Gal3 expression is markedly upregulated in the central nervous system (CNS), primarily due to its robust production by reactive microglia and macrophages under various neurological disease conditions (Lozinski et al. 2024). Gal3 critically mediates NLRX1‐driven lysosomal dysfunction. Through exacerbating the suppression of mTOR/TFEB signaling, it disrupts microglial clearance of NETs and amplifies neuroinflammation during cerebral ischemia‐reperfusion injury (Peng et al. 2025). As a vital pro‐apoptotic hub, Lgals3 cooperates with Bax to trigger mitochondrial damage and neuronal apoptosis in spinal cord injury (Ren et al. 2019; Mostacada et al. 2015). This process occurs via disruption of mitochondrial quality control under oxidative stress. In addition, Lgals3 acts as a key molecular target of zinc ions. Inhibition of the Lgals3‐Bax pathway maintains mitochondrial integrity and mitigates PANoptosis, which accounts for the organelle‐targeted neuroprotective effects of zinc (Bai et al. 2024). The loss of Lgals3 has been demonstrated to have potential in treating myocardial injury after IR, as Lgals3 inhibition reduced infarct size, cardiomyocyte apoptosis, and myocardial inflammation (Ibarrola et al. 2019; Mo et al. 2019). In cerebral IR injury mouse model, the deletion of Lgals3 has been implied to relieve the inflammatory reaction in brain tissues; some drugs exert a palliative effect in cerebral IR injury by reducing the expression of Lgals3, including attenuating neuronal apoptosis, improving neurological function, reducing brain infarct volume, and relieving brain tissue damage and inflammation (Y. Wang et al. 2021; X. Liu et al. 2019). Y. Wang et al. (2021) demonstrated that Lgals3 acts as a central regulator of inflammatory responses. Among core proteins modulated by QiShenYiQi, Lgals3 shows the highest interaction connectivity alongside albumin.

This study confirmed that Lgals3 expression was upregulated in mouse brain tissues following cerebral IR injury. The deletion of Lgals3 reduced infarct size, improved neurological function, and mitigated neuronal apoptosis and neuroinflammation in mice with cerebral IR injury. We selected systemic tail vein injection of AAV‐shLgals3 based on its ability to penetrate the blood‐brain barrier, the rationale for targeting the peripheral immune system involved in cerebral IR injury, and technical feasibility (Hwang et al. 2024). Although this method is not cell‐type specific, the prominent neuroprotective effects observed strongly validate Lgals3 as a promising therapeutic target. Further investigations using cell‐specific strategies are required to elucidate the exact underlying mechanisms.

Neuronal apoptosis is a major contributor to neurological deficits following cerebral IR injury (Li et al. 2024). Our results showed that Lgals3 knockdown alleviated neuronal apoptosis, increased the expression of Bcl‐2 protein, and repressed the expression of Bax, Cleaved caspase‐3, and Cleaved PARP proteins. The down‐modulation of anti‐apoptotic Bcl‐2 and the up‐modulation of pro‐apoptotic Bax, Cleaved caspase‐3, and Cleaved PARP have been reported in some brain disorders, including cerebral IR injury (D. Zhang et al. 2025). Thus, the loss of Lgals3 might mitigate neuronal apoptosis by regulating the expression of these apoptosis‐related proteins. Further, the severe inflammatory response during reperfusion process can cause more severe brain tissue damage and neurological impairment (Z. Cheng, Tu, et al. 2024). IL‐6, TNF‐α, and IL‐1β are common pro‐inflammatory factors, and IL‐4 is anti‐inflammatory factor; the enhanced release of IL‐6, TNF‐α, and IL‐1β but reduced IL‐4 levels have been discovered in the brain tissues of cerebral IR injury mice (Fang et al. 2024). Similarly, elevated IL‐6, TNF‐α, and IL‐1β levels and a decreased IL‐4 level were observed in the brain tissues of cerebral IR injury mice. Intriguingly, Lgals3 deletion reduced the secretion of IL‐6, TNF‐α, and IL‐1β, but intensified the secretion of IL‐4 in brain tissues of mice with cerebral IR injury. Consequently, Lgals3 deficiency may mitigate neuroinflammation in cerebral IR injury mice through dual modulation: suppressing pro‐inflammatory cytokines (IL‐6, TNF‐α, and IL‐1β) and elevating anti‐inflammatory mediators (IL‐4).

In this study, we detected significant activation of the TLR4/MyD88/NF‐κB signaling pathway in cerebral IR injury mouse brain tissues. Notably, Lgals3 knockdown significantly inhibited this pathway by reducing the expression of key components including TLR4, MyD88, p‐p65, and p65. As an inflammatory signaling receptor, TLR4 has been demonstrated to induce the inflammatory cascade response following vital organ IR injury via mediating the activation of the MyD88/NF‐κB signaling (S. Zhang et al. 2024). Available data have demonstrated that the activated TLR4/MyD88/NF‐κB pathway can trigger the secretion of pro‐inflammatory factors and further induce the degeneration and apoptosis of neurons (T. Ye et al. 2024). In cerebral IR injury, an inactivated TLR4/MyD88/NF‐κB pathway can relieve the inflammatory response and apoptosis to exert the neuroprotective function (Xu et al. 2024). A previous report has revealed that the repression of Lgals3 could mitigate the inflammatory responses in microglia by blocking the activation of the TLR4/MyD88/NF‐κB pathway (Y. Liu et al. 2022). Collectively, these findings indicate that Lgals3 deficiency ameliorates cerebral IR injury through inhibiting the TLR4/MyD88/NF‐κB signaling pathway. Notably, this study performed systemic Lgals3 knockdown in mice via tail vein injection of AAV9‐shRNA, without cell‐type‐specific gene manipulation. Given that the TLR4/MyD88/NF‐κB signaling pathway is mainly responsible for driving the pro‐inflammatory activation of microglia during cerebral ischemia‐reperfusion injury, and our results confirmed the direct interaction between Lgals3 and TLR4, we infer that the downregulation of Lgals3 alleviates neuronal apoptosis predominantly through an indirect mechanism: inhibiting microglia‐mediated neuroinflammation and subsequent inflammatory damage to neurons. Nevertheless, we cannot fully rule out the possibility that Lgals3 may directly modulate the intrinsic apoptotic pathway in neurons. The cell‐type‐specific role of Lgals3 and the precise downstream cascade in cerebral IR injury need to be further clarified by microglia‐ or neuron‐specific conditional gene knockout models in future research.

The current study primarily relies on a gene knockdown strategy and highlights that incorporating overexpression or rescue experiments would provide more compelling evidence for the functional role of Lgals3 in ischemia/reperfusion injury. Moreover, our study did not perform rescue experiments involving MyD88 overexpression or constitutive NF‐κB activation in Lgals3‐silenced cells to directly validate this pathway as the critical downstream event mediating Lgals3‐induced injury. However, the direct Lgals3‐TLR4 interaction confirmed by Co‐IP provides indirect support for the pathway mechanism, though this experimental limitation requires resolution in future investigations. Given the established consensus in the literature that Lgals3 is preferentially expressed in activated microglia during neuroinflammation (Stratoulias et al. 2023; Zhou et al. 2022), the protective effects observed in this study may primarily stem from the regulation of microglial function. Future studies should further validate these findings using cell‐specific knockout models.

This study identifies Lgals3 as a promising therapeutic target for ischemic stroke, given that its silencing significantly alleviated brain injury and improved functional recovery in preclinical models. Mechanistically, targeting Lgals3 offers a novel strategy to modulate the neuroinflammatory response by specifically inactivating the TLR4/MyD88/NF‐κB pathway, shifting the balance from pro‐inflammatory to anti‐inflammatory cytokines. These findings lay the groundwork for developing Lgals3‐based diagnostics and targeted therapies to improve outcomes in stroke patients.

## 5 Conclusion

In summary, this work demonstrated that upregulated Lgals3 in brain tissues of mice with cerebral IR injury. More importantly, the deletion of Lgals3 could relieve cerebral IR injury, as Lgals3 silencing reduced infarct volume, improved ameliorated neurological function, and relieved neuronal apoptosis and neuroinflammation in mice with cerebral IR injury. Moreover, the loss of Lgals3 suppressed the activity of the TLR4/MyD88/NF‐κB pathway. The present findings suggest that Lgals3 is closely involved in the progression of cerebral IR injury at the preclinical experimental level.

## Author Contributions

**Zhaodan Gan**: methodology, formal analysis, validation, Writing – original draft, resources, conceptualization. **Wen Zhang**: methodology, project administration, supervision, writing – review and editing, validation. **Jun Lu**: visualization, formal analysis, writing – review and editing, resources. **Jianqiu Xiao**: methodology, supervision, project administration, writing – review and editing, validation. **Yun Yang**: data curation, software, investigation, writing – review and editing. **Guangxu Xu**: methodology, project administration, validation, writing – review and editing, supervision. **Haifang Lai**: conceptualization, methodology, validation, formal analysis, resources, writing – original draft.

## Funding

The authors have nothing to report.

## Ethics Statement

This study adhered to the tenets of the Declaration of Helsinki and was approved by the Ethics Committee of The First Affiliated Hospital of Nanjing Medical University. The animal experiments were also approved by the Ethics Committee of The First Affiliated Hospital of Nanjing Medical University. All methods were carried out in accordance with relevant guidelines and regulations. All methods are reported in accordance with ARRIVE guidelines.

## Conflicts of Interest

The authors declare no conflicts of interest.

## Supporting information




**Supplementary figure 1**: Deducing batch effects and PCA analysis. (A) De‐batch correction of the differential expressed genes. (B) PCA analysis of the differential expressed genes. PCA: principal component analysis.

## Data Availability

The datasets generated and/or analyzed during the current study are available in the GEO database, including GSE97537 dataset (https://ftp.ncbi.nlm.nih.gov/geo/series/GSE97nnn/GSE97537/matrix/), GSE107983 dataset (https://ftp.ncbi.nlm.nih.gov/geo/series/GSE107nnn/GSE107983/matrix/) and GSE166162 dataset (https://ftp.ncbi.nlm.nih.gov/geo/series/GSE166nnn/GSE166162/matrix/).
